# Influence of Lineage-Negative Stem Cell Therapy on Articulatory Functions in ALS Patients

**DOI:** 10.1155/2019/7213854

**Published:** 2019-06-02

**Authors:** Wioletta Pawlukowska, Bartłomiej Baumert, Monika Gołąb-Janowska, Anna Sobuś, Agnieszka Wełnicka, Agnieszka Meller, Karolina Machowska-Sempruch, Alicja Zawiślak, Karolina Łuczkowska, Sławomir Milczarek, Bogumiła Osękowska, Edyta Paczkowska, Iwona Rotter, Przemysław Nowacki, Bogusław Machaliński

**Affiliations:** ^1^Department of Medical Rehabilitation and Clinical Physiotherapy, Pomeranian Medical University, Szczecin, Poland; ^2^Department of Neurology, Pomeranian Medical University, Szczecin, Poland; ^3^Department of General Pathology, Pomeranian Medical University, Szczecin, Poland

## Abstract

**Introduction:**

Amyotrophic lateral sclerosis (ALS) is a fatal, neurodegenerative disease, leading to loss of muscle strength and motor control. Impaired speech and swallowing lower the quality of life and consequently may induce acute respiratory failure. Bone marrow-derived stem and progenitor cells (SPCs) may be a valuable source of trophic factors. In this study, we assessed whether adjuvant cellular therapy could affect the levels of selected neurotrophins and proinflammatory factors in the cerebrospinal fluid (CSF) and subsequently prevent the deterioration of articulation.

**Materials and Methods:**

The study group consisted of 32 patients with sporadic ALS who underwent autologous lineage-negative (Lin^−^) stem cell intrathecal administration to the spinal canal. Lin^−^ cells were aspirated from the bone marrow and isolated using immunomagnetic beads and a lineage cell depletion kit. Patients were examined for articulatory functions by means of the Voice Handicap Index (VHI) questionnaire and Frenchay Dysarthria Assessment (FDA). In parallel, we carried out the analysis of selected trophic and proinflammatory factors in CSF utilizing multiplex fluorescent bead-based immunoassays.

**Results:**

Of the 32 patients who received the Lin^−^ progenitor cell therapy, 6 (group I) showed improvement in articulatory functions, 23 remained stable (group II), and 3 deteriorated (group III) on the 28^th^ day. The improvement was particularly noticeable in a better cough reflex, laryngeal time, and dribble reflex. A statistically significant lower level of brain-derived neurotrophic factor (BDNF) was observed on day 0 in group I compared to group II. The CSF concentrations of C-reactive protein (CRP) in group I significantly decreased 7 days after Lin^−^ SPC transplantation. On the contrary, a significant increase in the tumor necrosis factor receptor (TNF-R) level was confirmed among patients from group I with improvement of dribble and coughing reflex, tongue movements, and respiration on the 7^th^ day, as well as on day 28 including dribble reflex solely.

**Conclusions:**

An application of Lin^−^ stem cells could potentate the beneficial humoral effect. The prevention of deterioration of articulatory functions in ALS patients after applying adjuvant Lin^−^ stem cell therapy seems to be promising. Although the procedure is safe and feasible, it requires further in-depth studies.

## 1. Introduction

Amyotrophic lateral sclerosis is a neurodegenerative disease which causes progressive muscle weakness and loss of motor control [1]. Speech deterioration occurs as a prodromal symptom in about 25-30% of ALS sufferers, who display lower speech volume, imprecise articulation, or difficulties in raising the voice, screaming, or singing. Repetitive movements of the lips, tongue, and pharynx become slower due to a decline in the range of motion within those organs [2]. The time between the occurrence of the first signs of speech deterioration and a definitive diagnosis of ALS varies between 33 and 60 months [3]. Speech impairments in ALS manifest themselves in the form of flaccid, spastic, or mixed flaccid-spastic dysarthria [4]. Dysarthric disorders in ALS affect articulation, phonation, breathing, prosody, and resonance [5, 6]. Bulbar dysarthria is characterized by the most rapid progressive changes which first affect the tongue muscles and the orbicularis oris to subsequently impair the musculature of the soft palate, mandible, and pharynx. The facial and laryngeal muscles become dysfunctional last [7, 8]. Spastic and mixed flaccid-spastic dysarthria in ALS manifest themselves in distorted articulation, disturbed prosody, spastic dysphonia, and decreased respiratory capacity while speaking [9]. Other symptoms include monopitch voice, short phrases, distorted vowels, monoloudness, and “breathy” voice quality [10].

There is no effective treatment for dysarthria in ALS. Some symptomatic and compensatory therapies used today bring only temporary improvement in communication and raise quality of life in ALS patients [11–13]. Pharmacological treatment is limited with only class IV evidence. Sometimes patients with spastic dysarthria are temporarily helped by sucking ice, having it placed over the larynx, or taking antispastic drugs such as baclofen [14]. Botulinum toxin type A has been reported as effective in spastic dysarthria [15] and spasmodic dysphonia [16, 17]. Pyridostigmine may bring a temporary effect in some patients [18]. Another method of therapy is a palatal lift. The procedure may temporarily improve resonance by raising the weak soft palate to the level of normal palatal elevation and thus reduce hypernasality and hypophonia [19, 20]. A palatal augmentation prosthesis may also temporarily enhance articulation by lowering the palate, which improves the production of the lingual consonant sounds [20, 21]. There is no solid evidence as to how effective these prostheses are or how long they remain effective [22, 23].

The autologous bone marrow stem cell application might prove an effective alternative to classic ALS treatments. Stem cells seem to be a reasonable and promising option, all the more so since the intrathecal administration of neurotrophic factors alone failed to bring expected results in clinical trials [24] mainly due to their low bioavailability and very short half-life in the spinal canal. The administration of autologous stem/progenitor cells (SPCs) to individuals with neurodegenerative diseases is expected to result in trophic support for the host's neurons; slowdown in the progression of the disease, stimulating the secretion of deficient neurotransmitters; and differentiation into oligodendrocyte progenitor cells or neurons [25]. Since the 1960s, when the first neurotrophic factors were discovered and defined, there has been a steady increase in the research dedicated to neurotrophic protective factors, with a particular focus on “classical” brain-derived neurotrophins (NTs): nerve growth factor-beta (NGF-*β*), brain-derived neurotrophic factor (BDNF), and neurotrophin-3 (NT-3) [26, 27].

Apart from having a crucial neuroprotective effect, BDNF was also found to play an important role in neuronal survival and growth. It also serves as a neurotransmitter modulator [28, 29]. As most NTs present in body fluids have a very short half-life, any attempted treatments with recombined factors would be costly and the effects short-lived. Repeated cell injections performed at short intervals or administration of genetically modified stem cells would ensure prolonged and steady secretion of the desired soluble neurotrophic agents and create an appropriate microenvironment for the neuroregenerative processes [30]. First reports regarding the effects of such a pathophysiological compilation following the delivery of BDNF from genetically modified mesenchymal stem cells with overexpressed NTs are promising [31]. It has been recently shown that neuroinflammation mediated by glial cells and systemic immune activation may be a key factor contributing to the progression of ALS through mechanisms that can be either neuroprotective or neurodegenerating, depending on the type of cells and the motor neuron compartment involved [32, 33]. It has also been declared that ALS patients present signs of systemic inflammation, reflected in increased levels of CRP, tumor necrosis factor-alpha (TNF-*α*), interleukins (IL-6, IL-8), interferon-beta (IFN-*β*), and complement components such as C3 and C4 [34–36]. In ALS patients, CRP could be produced not only by the liver but also locally in the brain [37]. Moreover, the CRP level correlates with neurologic functional impairment and survival [38]. A meta-analysis by Hu et al. [39] provided a systematic review of 25 publications regarding blood inflammatory cytokines in ALS patients vs. control subjects. Results showed that the levels of TNF-*α*, TNF receptor 1, IL-6, IL-1*β*, IL-8, and vascular endothelial growth factor (VEGF) were significantly higher in ALS patients compared to controls, suggesting that these peripheral inflammatory cytokines might be biomarkers for ALS.

Lineage-negative cells are a very rare population of cells compared to mononuclear cells (MNCs) or mesenchymal stem cells. However, they are highly enriched by immature SPCs, including CD34^+^ cells, CD133^+^ cells, and expressing markers involved in migration, adhesion, and homing to the bone marrow and sites of tissue injury [27]. We have previously shown that umbilical cord blood- (UCB-) derived Lin^−^ cells strongly and specifically express classical NTs like BDNF, NGF, NT-3, NT-4, and the novel neurotrophic cytokines, as well as VEGF [27]. NT expression in the Lin^−^ population was much higher than in unsorted nucleated cells (NCs) [27]. We have also shown that these secreted factors support neuronal proliferation and *in vitro* survival in a conditioned medium from Lin^−^ SPCs [27]. The influence of intrathecal administration of Lin^−^ cells on trophic factors and proinflammatory factors in patients with ALS has been the subject of our previous published study [26].

In this consecutive study, we aimed to investigate whether intrathecal administration of autologous bone marrow-derived Lin^−^ cells can lead to the prevention of deterioration of articulatory functions. Additionally, we used functional scales (Norris and ALS Functional Rating Scale) to assess the overall neurological condition of ALS patients and confront it with the objective evaluation of articulation. Because growth factors play a pivotal role in the regeneration and SPCs can release a number of growth factors, we hypothesized that adjuvant stem cell-based therapy could also bring specific changes in various neurotrophins and proinflammatory factors profiles in the CSF of ALS patients.

## 2. Material and Methods

### 2.1. Subjects

The study was designed as a prospective, open-label, nonrandomized clinical trial in a single center for subjects with ALS. The trial (international number: NCT02193893) was approved by the Ethics Committee of the Pomeranian Medical University in Szczecin and conducted in accordance with the Declaration of Helsinki [26]. Prior written informed consent was obtained from all of the subjects. Patients enrolled in the study met the following criteria:

- Under 65 years of age
- The diagnosis of a probable or certain sporadic ALS form based on the El Escorial Revised Criteria
- Ability to express informed consent
- Observation of the course of riluzole-controlled disease for 3 months preceding the use of cell therapy
- Mild to moderate disability documented by satisfactory bulbar and spinal motor functions (minimum score 3 on the ALS-FRSr scale for swallowing and 2 points for food preparation and walking)
- Forced vital capacity (FVC) result greater than or equal to 50%
- Without cancer, signs of inflammation, diabetes, cardiovascular disease, chronic kidney, and liver disease, in euthyreosis, not receiving drugs that could affect stem cell activity

The study enrolled 32 patients—sixteen females and sixteen males—aged between 27 and 65 years (mean: 53.8 ± 8.17) with sporadic ALS [26] according to the El Escorial Revised Criteria [40]. The patients with predicted survival time of over 12 months established on the basis of the general and neurological condition were administered Lin^−^ SPCs during their stay in the Department of Neurology of the Pomeranian Medical University in Szczecin.

The outcome measures were as follows:

- *Primary outcome measures:* safety of autologous bone marrow Lin^−^ stem/progenitor cell infusion in enrolled patients
- *Secondary outcome measures:* efficacy of autologous bone marrow Lin^−^ stem/progenitor cell infusion in enrolled patients

A 3-month period following the enrollment was dedicated to natural history observation, during which controlled administration of riluzole was continued. Patients over 65 were excluded from the study, as it had been previously demonstrated that cell growth of expanded *in vitro* stem cells is strictly related to the donor's age [41]. Patients with evidence of any concurrent illness or receiving any medications (including potentially other previously applied stem cell-based therapies) which might affect bone marrow were also excluded.

#### 2.1.1. Speech Test: VHI Questionnaire

International research projects have demonstrated that the Voice Handicap Index (VHI) questionnaire is a reliable tool for the subjective evaluation of voice in ALS patients and that the scores it provides are consistent. The 30-item VHI questionnaire comprising 3 subscales of 10 items each—the physical (P) items, functional (F) items, and emotional (E) items—is a recommended method of subjective assessment of the severity of speech disorders by ALS patients themselves. Cronbach's alpha for the entire cohort was 0.95, indicating high internal consistency of the 30 items. The VHI is a reliable and valid tool that can be recommended for ALS. The questionnaire is widely used throughout the world in a number of language versions including Persian, Croatian, Italian, Brazil, Latvian, Greek, and Polish [42–47]. The subjects underwent the assessment on days 0, 7, and 28 following the Lin^−^ cell administration.

#### 2.1.2. Speech Test: FDA

One of the most important objective tests for evaluating articulation organs is the Frenchay Dysarthria Assessment (FDA). The FDA is a standardized test which relies on a 9-point rating scale applied to a patient. It provides information based on the observation of oral structures, functions, and speech. The test evaluates the following functions: reflexes, respiration, tongue, lips, the soft palate, laryngeal, and intelligibility. A 5-point rating scale (a–e) is used for the assessment, where letter “a” represents norm, “b” mild severity, “c” moderate, “d” considerable severity, and “e” profound severity. FDA is also used to assess the severity of the articulatory organ disorders and to monitor the effects of treatment [48]. The test was conducted by a clinical speech therapist with 12 years' experience of treating neurological conditions, predominantly Parkinson's disease. The second edition of FDA utilizes the latest findings concerning motor speech disorders and their contribution to neurological diagnosis. It has good feasibility (missing data < 5%), a high reliability of the total score (0.94), an excellent interrater agreement for the total score (0.96), and moderate to large construct validity for 81% of its items [48]. The subjects underwent the assessment on days 0, 7, and 28 following the Lin^−^ cell administration. Based on a detailed analysis of the FDA scores, the patients were divided into 3 groups: group I—comprising patients who demonstrated improvement of the articulation organs, group II—patients whose articulation remained stable, and group III—patients with deterioration.

#### 2.1.3. Neurological Assessment

Assessment of disease progression was performed using two functional ALS scales—the Norris scale and the revised ALS Functional Rating Scale—on 0 (on the day of bone marrow collection and Lin^−^ cell administration), 7, and 28 days after cell application. The Norris scale describes limb and bulbar functions. The limb scale evaluates 21 items and the bulbar scale 13. Each item is rated in four ordinal categories [49]. Revised ALS-FRS is based on a questionnaire which allows the assessment of physical functions in activities of daily living, and it is the most widely used system for the functional rating of ALS patients. It is divided into four clinical domains: (1) bulbar function, (2) fine motor function, (3) gross motor function, and (4) respiratory function. Each section includes 3 questions scored from 0—loss of function, to 4—normal function. Total score ranges from 0 to 48 [50].

### 2.2. Cells

Bone marrow (BM) was harvested from the patients after we had obtained their written informed consent. BM samples (100-120 ml) were aspirated in local anesthesia from the posterior iliac crest of the recruited patients and subsequently resuspended in collecting the medium (phosphate-buffered saline, pH 7.2) and heparin (20 U/ml; Gibco, USA). Bone marrow samples were lysed in BD Pharm Lyse lysing solution (BD Biosciences, San Jose, CA, USA) for 15 min at room temperature in the dark and washed twice in phosphate-buffered saline (PBS). The obtained suspension of BM nucleated cells (NCs) was subjected to immunomagnetic separation procedures. Isolation procedures were performed according to the manufacturer's instructions, according to the GMP conditions. Briefly, lineage-negative cells were isolated through negative selection using a MidiMACS separator (Miltenyi Biotec, Auburn, CA, USA). To isolate a lineage-negative cell population, a Lineage Cell Depletion Kit (Miltenyi Biotec, Auburn, CA, USA) was used. One hundred microliters of biotin-antibody cocktail recognizing the lineage-specific cell antigens was added per 10^8^ cells according to the manufacturer's recommendations. After washing in PBS, 100 *μ*l of Anti-Biotin MicroBeads for magnetic cell labeling was added. Labeled cell suspension was loaded onto a MACS LS column (Miltenyi Biotec), and unlabeled cells passing through the column were collected (Lin^−^) [27]. Before administration, the cells were maintained in 2 ml of sterile PBS.

After isolation, lineage-negative cells were resuspended in 100 *μ*l PBS and stained with mouse anti-human monoclonal antibodies for the following SPC markers: CD34 conjugated with allophycocyanin (APC) (BD Biosciences, San Jose, CA, USA), CD133 conjugated with phycoerythrin (PE) (Miltenyi Biotec, Auburn, CA, USA), and CD144 (FITC) (BD Biosciences, San Jose, CA, USA). After incubation for 20 min at 4°C, the cells were washed twice in PBS. Cell fluorescence was measured, and the data were analyzed using a fluorescence-activated cell analyzer (LSRII, BD Biosciences, San Jose, CA, USA) and BD FACSDiva software. Typically, 10,000 events were acquired to determine the percentage of a subpopulation within the lineage-negative SPCs. Populations of hematopoietic SPCs based on CD34^+^ and CD133^+^ expressions, early CD34^+^/CD133^+^/CD144^+^ endothelial progenitor cells (EPCs), and endothelial CD144^+^ cells were analyzed [27].

### 2.3. Administration Procedure

The total isolated Lin^−^ SPCs were slowly administered into the subarachnoid space via lumbar puncture (between vertebrae L3-L4 or L4-L5). The number of administered Lin^−^ cells varied among patients (mean: 6.26 × 10^6^ ± 5.84). After the injection, the patients were advised to maintain the supine position for at least 24 hours.

### 2.4. Molecular Analysis: Multiplex Assay

Concentrations of selected factors (TNF-*α*, TNF-R, CRP, BDNF, NT-3, and NGF-*β*) were assessed in CSF using multiplex fluorescent bead-based immunoassays (Luminex Corporation, Austin, TX, USA). Analyzed CSF samples were collected on the day of the cell injection (day 0) and on the 7^th^ day. Assays were performed according to the manufacturer's protocol, as described previously [51].

### 2.5. Statistical Analysis

The statistical null hypothesis we tested was that the intrathecal administration of autologous BM-derived Lin^−^ cells cannot lead to the prevention of deterioration of articulation. The primary objective of this investigation was to analyze the alternative hypothesis that intrathecal administration of autologous BM-derived Lin^−^ cells can lead to the prevention of deterioration of articulation. We also hypothesized that adjuvant cell therapy could also bring specific changes in various neurotrophins and proinflammatory factor profiles in CSF of ALS patients. To assess the equality of variances for variables, Levene's test was used before a comparison of means. The test has shown significance (*p* < 0.05). For this reason and because of the nonnormality of the distributions between variables (Shapiro–Wilk test), the numerical data were compared between groups using the nonparametric Friedman analysis of variance by ranks for more than two groups of repeated variables. The occurrence of nominative clinical data was compared between groups by means of the chi-squared test or Yates' chi-squared test if needed. *p* < 0.05 was considered to indicate statistical significance. All statistical analyses were performed with STATISTICA 12.5 PL.

## 3. Results

32 patients suffering from sporadic ALS (sixteen females and sixteen males) were included in the study. All of them were administered autologous bone marrow-derived Lin^−^ cells injected intrathecally. A thorough analysis of a fraction of Lin^−^ cells in our earlier studies revealed its composition as a heterogenic population of cells consisting of precursor cells, progenitor cells, and stem cells [27]. By employing flow cytometry, we had previously shown that Lin^−^ cells contain populations of CD34^+^ cells (12.1% ± 7.2), CD133^+^ cells (12.3% ± 8.2), endothelial progenitor cells (CD34^+^, CD133^+^, and CD144^+^ cells, 1.7% ± 1.1), and cells with the mesenchymal stem cell phenotype (CD105^+^, CD73^+^, CD90^+^, CD45^−^, CD34^−^, CD11b^−^, CD19^−^, and HLA-DR^−^ cells, 0.0084% ± 0.0108) [27]. FDA and VHI scales were used for the objective and subjective evaluations of the patients' articulation functions, respectively, performed at baseline, 7 days, and 28 days following the cell administration. The Norris scale and revised ALS Functional Rating Scale were used for the functional evaluation of enrolled patients, performed similarly at baseline, 7 days, and 28 days following the cell infusion.

### 3.1. Speech Tests and Neurological Evaluation

Of the 32 patients who received the Lin^−^ stem/progenitor cell therapy, 6 (group I) showed improvement in articulatory functions, 23 remained stable (group II), and 3 deteriorated (group III) on the 28^th^ day according to the objective FDA scale.  Table 1 shows the comparison of the initial anthropometric parameters of the groups. Group I subjects were older than their group II counterparts, and the difference in age was statistically significant (*p* = 0.021). No notable differences were recorded between the three groups regarding the quantities of administered Lin^−^ cells and sex of the patients. The patients from group I had a tendency towards a longer interval from symptom onset to diagnosis, although statistically insignificant. However, this factor is considered as a marker of favorable prognosis [52].

Additionally, we confronted articulation according to the FDA scale with functional condition based on two scales—Norris and ALS-FRSr. Assessment was performed on 0, 7, and 28 days after infusion of Lin^−^ cells. The results, although not statistically significant, were consistent with those obtained from the evaluation of the articulation. The ALS-FRSr and Norris scale scores increased slightly on the 28^th^ day among patients from groups I and II. Group III patients deteriorated according to ALS-FRSr and remained unaffected according to the Norris scale. It is interesting to note that the patients from group III with the deterioration of articulation initially had the highest score in Norris scales and ALS-FRSr scales. The described changes are presented in Table 1.

Table 2 shows the efficiency of the articulation organ functions in 32 ALS patients divided into 3 groups evaluated at different time points (0, 7, and 28 days). The most significant improvement was noticed on the 7^th^ day following the cell administration in respect of cough reflex (34%), laryngeal time (32%), and dribble/drool reflex (28%). The improvement, though, was short-lasting and was no longer observed on the 28^th^ day after the cell infusion.

Table 3 illustrates the subjective assessment of articulation based on the VHI questionnaire carried out by 32 ALS patients divided into 3 groups at different time intervals following the Lin^−^ cell administration. On the 7^th^ day, 53% of the subjects reported improvement in articulation. On the 28^th^ day, the figure grew to 62.5% and 9.3% did not feel any change in their speech, whereas 28% of the patients claimed that in their case, speech/articulation had actually deteriorated. Statistically, significant correlation was observed between the baseline FDA and VHI scores and those obtained on day 7 following the application of Lin^−^ cells (*p* = 0.0128).

### 3.2. Molecular Analysis

**Figure 1:**
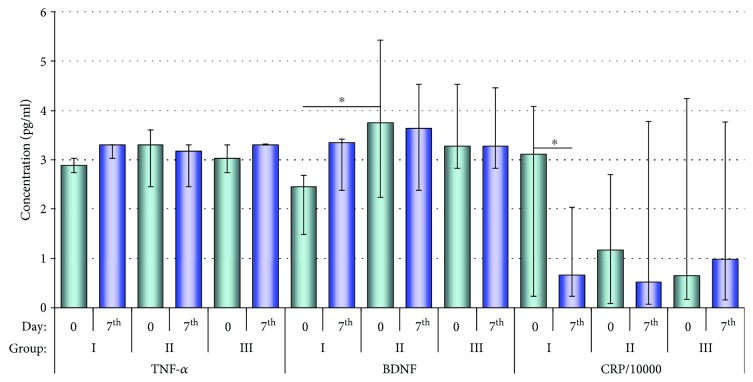
The CSF levels of TNF-*α*, BDNF, and CRP in different groups (0 and 7^th^ days). Data are presented as median (interquartile range); ^∗^*p* < 0.05.

Using Luminex multiplex assay, we have investigated concentrations of CRP, TNF-*α*, TNF-R, BDNF, NGF-*β*, and NT-3 in CSF on day 0 and the 7^th^ day post-cell infusion. The subjects with improved articulation objectively established by means of FDA (group I) demonstrated a statistically significant decrease in CRP levels during the first seven days after transplantation (*p* = 0.043). The analysis of FDA scores recorded in groups I and II (improvement vs. no improvement) on day 0 revealed a significantly lower BDNF level in group I compared to group II (*p* = 0.028). Besides, we noticed a tendency to increase the level of BDNF (*p* = 0.14) and TNF-*α* (*p* = 0.068) on the 7^th^ day from the application of the Lin^−^ cells in group I. A statistically significant correlation was also found in groups I and II between the patients' age and their FDA scores obtained on days 0–7 (*p* = 0.043)—improved articulation was recorded in statistically older subjects. Changes in concentrations of proinflammatory factors and neurotrophins in CSF are presented in Table 4 and Figure 1 (selected factors).

Analysis of particular functions and efficiency of the articulation organs using the FDA scale revealed a statistically significant increase in the concentration of TNF-R in group I on the 7^th^ day following the Lin^−^ administration and improvement in the following: dribble reflex (*p* = 0.018), cough reflex (*p* = 0.015), tongue movements (*p* = 0.015), and respiration (*p* = 0.046). Moreover, group I subjects with improved dribble reflex demonstrated a significantly higher TNF-R concentration (*p* = 0.036) on day 28, while on the same day, group III patients with declined postinfusion laryngeal time showed a statistically significant increase in the concentration of TNF-*α* in CSF (*p* = 0.043).

## 4. Discussion

ALS is classified as sporadic (90 to 95% of the diagnosed cases), of unknown etiology, and familiar [53]. It is believed that there are several factors that may contribute to the onset of the disease, such as excitotoxicity by neurotransmitter glutamate, accumulation of neurofilaments, deficiency of neurotrophic factors, immunity changes, physical trauma, and persistent viral infections, as well as chemical and physical environmental factors [54]. With the progress of the disease, communication difficulties increase while sentences become simpler and shorter, culminating, in advanced phases, to answering questions through the use of keywords or “yes/no” answers [55]. These changes, together with the loss of functional independence caused by ALS, lead to an extremely discouraging situation for the individual.

The assumed effectiveness of Lin^−^ stem cell therapy for ALS is based on SPCs' ability to produce growth factors and cytokines that exert neuroprotective activity, thus providing supportive environment and protection for neurons [56]. In our earlier study, we demonstrated that the therapy is safe and feasible [26]. An autologous population of bone marrow-derived lineage-negative SPCs demonstrates higher long-term self-regeneration potential and capacity to produce neurotrophic and angiopoietic factors than other nucleated cells [48].

The potential of stem cell-based therapy has not yet been explored in the context of its employment for treatments aimed at ameliorating articulation in ALS patients. In the course of our study, we carried out the assessment of articulation disorders in ALS patients utilizing the subjective VHI questionnaire and the objective FDA scale. We also analyzed the effect of the adjuvant Lin^−^ stem cell administration on the articulatory functions and the CSF levels of neurotrophic and inflammatory factors. Our methodology was based on previous findings which demonstrated that the half-time of trophic factors, and thus the clinical effect, is limited to a few days following the cell infusion [26]. At the same time, we used functional scales (Norris and ALS-FRSr) to assess the overall neurological condition and confront it with the objective evaluation of articulation.

According to the FDA scale applied on the 28^th^ day, 6 of the 32 patients who had received the Lin^−^ SPCs showed slight improvement in articulatory functions, 23 remained stable, and in 3 others the functions deteriorated. However, the effects were transient and the observation time was short. The results, although not statistically significant between groups, were consistent with those obtained from the evaluation of the functional condition based on the ALS-FRSr and Norris scales. Patients with stabilization or with an inconsiderable improvement in articulation also showed a tendency to the improvement in the functional scales. Similarly, in the group of patients with progression of articulatory dysfunction, a slight deterioration according to the functional scales was also observed.

The noticeable improvement observed on the 7^th^ day after the cell infusion was seen in regard to cough reflex (34% of the subjects), laryngeal time (32%), and dribble/drool reflex (28%). Correlation between the objective FDA scores and the results of subjective VHI questionnaire could only be observed until the 7^th^ day following the procedure. Between days 7 and 28, the patients subjectively reported a deterioration of their articulatory functions, which did not reflect the positive FDA scores. Improved articulation based on the FDA scale was demonstrated in statistically older patients (*p* = 0.043).

Analysis of factors in CSF on the 7^th^ day following the Lin^−^ cell administration revealed a statistically significant correlation of the decrease in CRP concentration in patients with improved articulatory function. Interestingly, patients with the highest baseline CRP levels demonstrated the best response, whereas those with low baseline CRP concentration with a growing tendency showed clinical deterioration in their condition on the 7^th^ day.

The important role of neuroinflammation mediated by glial and immune cells in the pathogenesis of ALS is widely accepted [57]. Numerous inflammatory proteins have been previously shown to be altered by this disease [58, 59]. CRP has been described as a biomarker of the inflammatory response with significant prognostic value in a vast number of tumors and rheumatic and cardiovascular diseases [60]. CRP could be produced not only by the liver but also locally in the brain [37]. Keizman et al. have previously correlated CRP levels with neurological disability in ALS [61]. Lu et al. established that IL-6 associated with CRP levels is the only marker, which shows an increase in the expression toward the end-stage ALS [62]. A study by Nagel et al. on 289 ALS patients and 506 controls showed a moderate inverse correlation of CRP with the revised ALS Functional Rating Scale (ALS-FRSr) [63]. Our previous and current observation might lead to the conclusion that humoral therapy has a suppressive effect on inflammation [26]. However, as ALS is a disease of complex etiopathogenesis, the therapy does not always bring substantial clinical improvement. High baseline levels of proinflammatory factors may appear to be a positive prognostic indicator related to the ability to control its course.

Baseline BDNF concentration in CSF was significantly lower in patients who demonstrated improved articulation compared to those whose articulation had remained unchanged. The improvement in articulation was accompanied by a tendency toward higher levels of BDNF on the 7^th^ day after the cell infusion (*p* = 0.14).

Analysis of individual functions and efficiency of the articulatory organs carried out using the FDA scale on the 7^th^ day following the Lin^−^ cell administration showed a statistically significant increase in TNF-R concentration in the group I subjects who demonstrated improvement in dribble/drool reflex (*p* = 0.018), cough reflex (*p* = 0.015), tongue movements (*p* = 0.015), and respiration (*p* = 0.046). On top of that, on the 28^th^ day following the Lin^−^ application, we observed a considerable increase in the concentration of TNF-R in group I patients with improved dribble/drool reflex (*p* = 0.036). However, on the same day, group III subjects, with declined laryngeal time, showed a statistically significant increase in the levels of TNF-*α* in CSF (*p* = 0.043).

TNF-*α* can be synthesized and released in the brain by astrocytes and some populations of neurons. There is a robust and rapid increase in TNF-*α* expression levels in the CNS both after acute insults and in a number of chronic neurodegenerative disorders [64, 65]. Hattori et al. reported that TNF-*α* is involved in modulating neuronal cell function through an indirect mechanism by which it stimulates the synthesis and secretion of the nerve growth factor (NGF) in fibroblasts and glial cells [66]. TNF-*α* induces BDNF expression in astrocytes not only through the activation of NF-*κ*B but also via the activation of the C/EBP*β* transcription factor connected with the ERK MAP kinase pathway [67]. Based on various studies focused on the role of proinflammatory cytokines in the brain, TNF-*α* is usually regarded as an agent playing an important role in sustaining and modulating neurodegenerative events or sometimes promoting cell survival [68, 69]. Brambilla et al. identified TNF receptor 1 (TNFR1) signaling as a major promoter of glial cell line-derived neurotrophic factor (GDNF) synthesis/release from human and mouse spinal cord astrocytes *in vitro* and *in vivo* [70]. They also demonstrated that motor neuron loss is more pronounced in *SOD1^G93A^* mice lacking the TNFR1 at the late phase of the disease, i.e., when ALS progression is significantly accelerated [70].

Of the 32 ALS patients subjected to the Lin^−^ cell infusion, 11 demonstrated improved articulatory functions on the 7^th^ day, particularly noticeable in the cough reflex and laryngeal time (10 patients) and dribble/drool reflex (9 patients). Reduced or absent cough reflex in ALS sufferers is a serious abnormality inevitably leading to the accumulation of excessive saliva in the airways, which adversely affects respiration [71]. Secretions that obstruct the airways can result in a partial or complete collapse of the lung, contributing to acute or acute-on-chronic respiratory failure, which remains the most common cause of death in ALS [72, 73]. Maintaining an effective cough reflex could significantly contribute to the reduction of mortality and many serious infectious complications in patients with ALS.

In conclusion, our experimental adjuvant therapy with the application of autologous bone marrow-derived lineage-negative cells injected intrathecally proved entirely safe for ALS patients. No immediate or delayed, topical or systemic adverse events following the treatment were observed. During this short observation, some patients from the study deteriorated while others remained stable. The postapplication, short-termed improvement of articulatory functions, particularly the cough reflex, laryngeal time, and dribble reflex, observed in one third of the subjects, may provide valuable incentives for further comprehensive investigations. Establishing whether local neuroinflammation and CRP production could be direct prognostic factors or targets for therapy requires further studies, but our results seem to be promising.

## 5. Potential Study Limitations

Although our study provides valuable findings, there are a few limitations that need to be taken into account. The first one is a relatively small number of patients enrolled in the study, mainly limited by strict inclusion criteria. The second one refers to a considerable heterogeneity of the studied sample in respect of the age of onset of the symptoms, their duration, and the number of cells harvested and administered (mostly statistically insignificant). This heterogeneity, however, corresponds with typical key features of ALS. Another limitation concerns the fact that the study was carried out without the control group, which limits the possibility of drawing a direct conclusion regarding the effects of Lin^−^ cell application. In the case of a fatal, neurodegenerative disease such as ALS, it seems rather unethical to enroll patients without offering them a chance for potentially beneficial intervention.

Since this is still a preliminary study of such cell application, we must be cautious when interpreting the obtained data. Taking into consideration the complexity of ALS pathophysiology, it is still unclear whether the described correlations favor Lin^−^ cell administration as a beneficial treatment strategy for ALS or reflect other, unknown factors. Additionally, we can expect that the potential beneficial effects of humoral therapy in ALS patients may possibly be prolonged with repeated cell injections. The last drawback of the study is a short observation period. It is due to the fact that the study is being continued on a larger cohort of ALS subjects who undergo a cyclical Lin^−^ cell infusion during which they receive in total three injections and the levels of trophic and proinflammatory factors are measured at different time intervals.

## Figures and Tables

**Table 1:** Characteristic of the groups with ALS-FRSr and Norris scale results.

Characteristic	Group I (*n* = 6)	Group II (*n* = 23)	Group III (*n* = 3)	*p* value		
				I vs. II	I vs. III	II vs. III
Age (mean ± SD, years)	60.3 ± 3.82	51.8 ± 8.55	56.3 ± 3.05	0.021^a^	0.97^a^	0.89^a^
Sex (male/female)	4/2	11/12	1/2	0.72^b^	0.81^b^	0.89^b^
Time from symptom to diagnosis (mean ± SD, months)	40.8 ± 31.03	31.13 ± 27.62	26 ± 19.28	0.87^a^	0.92^a^	0.41^a^
Number of Lin^−^ cells administered (mean ± SD)	(6.47 ± 6.74) × 10^6^	(6.44 ± 6.11) × 10^6^	(4.5 ± 0.89) × 10^6^	0.51^a^	0.15^a^	0.21^a^
ALS-FRSr score (mean ± SD)						
Before Lin^−^ infusion	30 ± 5.4	26.9 ± 4.3	33 ± 5.6	0.63^c^	1.0^c^	0.17^c^
7 days after Lin^−^ infusion	30 ± 5.4	26.9 ± 4.3	33 ± 5.6	0.63^c^	1.0^c^	0.17^c^
28 days after Lin^−^ infusion	31.5 ± 6.4	27.7 ± 4.6	30.7 ± 7.4	0.58^c^	1.0^c^	0.28^c^
Norris scale score (mean ± SD)						
Before Lin^−^ infusion	84.5 ± 16.9	81.2 ± 15.6	97 ± 14.1	1.0^c^	0.47^c^	0.25^c^
7 days after Lin^−^ infusion	87.6 ± 14.1	81.2 ± 15.6	97 ± 14.1	1.0^c^	0.77^c^	0.19^c^
28 days after Lin^−^ infusion	92.5 ± 14.8	82.8 ± 15.5	97 ± 14.1	0.64^c^	1.0^c^	0.65^c^

^a^Friedman analysis of variance by ranks for more than two groups. ^b^Chi-squared test or Yates' chi-squared test. ^c^ANOVA Kruskal-Wallis test.

**Table 2:** Evaluation of articulation organ functions by FDA in 32 ALS patients divided into 3 groups (I—improved articulation, II—lack of improvement, and III—deteriorated articulation), measured 7 and 28 days after Lin^−^ cell administration compared to the baseline values (day 0).

	0 day vs. 7^th^ day			7^th^ day vs. 28^th^ day			0 day vs. 28^th^ day		
	Group I	Group II	Group III	Group I	Group II	Group III	Group I	Group II	Group III
Cough reflex	11	16	5	5	17	10	7	18	7
Swallow reflex	6	23	3	2	25	5	6	19	7
Dribble/drool reflex	9	20	3	6	20	6	10	19	3
Respiration	8	18	6	3	23	6	9	14	9
Lips	6	24	2	2	26	4	9	17	6
Soft palate	7	22	3	2	26	4	6	22	4
Laryngeal time	10	21	1	4	17	11	9	17	6
Laryngeal pitch	6	23	3	4	20	8	8	18	6
Laryngeal volume	8	20	4	3	23	6	9	18	5
Laryngeal in speech	7	21	4	4	22	6	8	19	5
Tongue	7	17	8	4	21	7	10	12	10
Intelligibility words	4	24	4	3	25	4	3	28	1
Intelligibility sentences	4	24	4	5	24	3	7	24	1
Intelligibility conversation	6	20	6	6	23	3	8	19	5

**Table 3:** Subjective evaluation of articulation improvement carried out by means of the VHI scale at different time intervals following Lin^−^ cell administration in 32 ALS patients divided into 3 groups according to FDA (I—improved articulation; II—lack of improvement; III—deteriorated articulation).

	0 day vs. 7^th^ day	7^th^ day vs. 28^th^ day	0 day vs. 28^th^ day
Group I	17	9	20
Group II	6	16	3
Group III	9	7	9

**Table 4:** The CSF concentrations of TNF-*α*, BDNF, NGF-*β*, CRP, NT-3, and TNF-R in groups I, II, and III (0 and 7^th^ days).

Concentration in CSF	Group I (*n* = 6)			Group II (*n* = 23)			Group III (*n* = 3)		
	Median (pg/ml)	Interquartile range	*p* value	Median (pg/ml)	Interquartile range	*p* value	Median (pg/ml)	Interquartile range	*p* value
TNF-*α* 0 d	2.88	0.29	0.068	3.31	0.58	0.409	3.026	0.58	0.180
TNF-*α* 7 d	3.31	0.29		3.17	0.29		3.32	0.00	

BDNF 0 d	2.45^∗^	0.45	0.144	3.75^∗^	2.17	0.262	3.27	1.71	0.990
BDNF 7 d	3.35	0.30		3.64	1.48		3.27	1.63	

NGF-*β* 0 d	0.74	0.20	0.500	0.84	0.20	0.948	0.95	0.16	0.109
NGF-*β* 7 d	0.84	0.20		0.84	0.20		0.74	0.00	

CRP 0 d	31104	30462	0.043	11667	24463	0.807	6472	40628	0.593
CRP 7 d	6680	16966		5144	34944		9862	36053	

NT-3 0 d	352.73	0.00	1.000	352.73	113.79	0.675	352.73	168.26	0.180
NT-3 7 d	352.73	54.47		352.73	107.39		352.73	233.68	

TNF-R 0 d	918.28	144.34	0.138	1006.18	190.83	0.115	1086.91	277.03	0.285
TNF-R 7 d	1176.05	335.68		1073.01	364.67		1070.30	318.57	

Data are expressed as median (interquartile range); *p* value—0 day vs. 7^th^ day; ^∗^*p* < 0.05 for difference between group I and II patients; Mann-Whitney *U* test.

## Data Availability

The data used to support the findings of this study are available from the corresponding author upon request.
